# A prospective study comparing water only with positive oral contrast in patients undergoing abdominal CT scan

**DOI:** 10.1038/s41598-020-63838-3

**Published:** 2020-04-22

**Authors:** Pascale A. M. de Wit, Jeroen A. W. Tielbeek, Pascal R. van Diepen, Ikrame Oulad Abdennabi, Ludo F. M. Beenen, Shandra Bipat

**Affiliations:** Amsterdam UMC, University of Amsterdam, Department of Radiology & Nuclear Medicine, Meibergdreef 9, 1105 AZ Amsterdam, The Netherlands

**Keywords:** Cancer screening, Gastric cancer, Hepatocellular carcinoma, Pancreatic cancer

## Abstract

Consecutive adults scheduled to undergo abdominal CT with oral contrast were asked to choose between 1000 ml water only or positive oral contrast (50 ml Télébrix-Gastro diluted in 950 ml water). Two abdominal radiologists independently reviewed each scan for image quality of the abdomen, the diagnostic confidence per system (gastrointestinalsystem/organs/peritoneum/retroperitoneum/lymph nodes) and overall diagnostic confidence to address the clinical question (not able/partial able/fully able). Radiation exposure was extracted from dose reports. Differences between both groups were evaluated by Student’s t-test, Mann-Whitney-U-test or chi-square-test. Of the 320participants, 233chose water only. All baseline characteristics, image quality of the abdomen and the diagnostic confidence of the organs were comparable between groups and both observers. Diagnostic confidence in the water only group was more commonly scored as less than good by observer1. The results were as follows: the gastrointestinal system(18/233vs1/87; p = 0.031), peritoneum (21/233vs1/87; p = 0.012), retroperitoneum (11/233vs0/87; p = 0.040) and lymph nodes (11/233vs0/87; p = 0.040). These structures were scored as comparable between both groups by observer2. The diagnostic confidence to address the clinical question could be partially addressed in 6/233 vs 0/87 patients (p = 0.259). The water only group showed a tendency towards less radiation exposure. In summary, most scan ratings were comparable between positive contrast and water only, but slightly favored positive oral contrast for one reader for some abdominal structures. Therefore, water only can replace positive oral contrast in the majority of the outpatients scheduled to undergo an abdominal CT.

## Introduction

### Background

CT protocols vary by institution, equipment, setting and clinical question. However, the default abdominal CT protocol for outpatients regularly includes both oral contrast (either positive or negative) and an intravenous contrast administration. The use of positive oral contrast in the outpatient setting has several direct and indirect effects, such as increased costs, decreased practice efficiency and patient inconvenience/discomfort. The latter has been shown by Harieaswar *et al*.; patients rated oral contrast significantly worse than intravenous cannulation and injection^1^. The question arose whether oral contrast can be eliminated. The justification of omitting oral contrast for emergency department patients has been questioned and extensively studied, leading to withholding oral contrast in these patients^2–5^. However, it is not clear whether the advantages of withholding oral contrast in the emergency department can be extrapolated to the outpatient setting, as they have different clinical questions and patient spectrum. There are no sufficient data on withholding oral contrast in this patient population^6–9^ and the available data are also equivocal.

### Positive and Negative Oral Contrast

However several studies^7–10^ evaluated the role of ‘’negative oral contrast” with water only and showed that the use of water only had similar image quality in follow-up abdominopelvic CT for general oncological indications^7–9^ and even better delineation^8,10^ and/or diagnosis compared to “positive oral contrast”. Although limited data is available, it seems that positive oral contrast can be replaced by water only, without losing image quality and the confidence of the diagnosis.

### Radiation Exposure

Another issue associated with positive oral contrast, is the radiation exposure. One study by Wang *et al*. showed higher radiation exposure for scans performed with positive oral contrast than those with negative oral contrast in phantoms, respectively 8.7 ± 0.1 mGy, and 8.2 ± 0.2 mGy (6.1% higher than in water only, p = 0.02). In patients these values were respectively 13.1 mGy and 11.8 mGy (11.0% higher than in water only p = 0.003)^11^.

However, all the aforementioned data^7–11^ were retrospectively obtained and it is known that retrospective studies have several limitations, such as selection bias, missing data and potential confounders. This makes the implementation of the findings of these studies in routine practice difficult.

### Pilot Study

In the Netherlands many hospitals still use Télébrix Gastro as oral contrast agent for general oncological/hematological indications.

Oral contrast has a predominant role in the evaluation of the gastrointestinal mucosa or bowel distension. However, the focus for most outpatient scans is not the gastrointestinal mucosa and/or bowel distension, but on the evaluation of visceral or metastatic disease. Therefore, intravenous contrast is necessary^12,13^. This is the reason we previously performed a single-centre, prospective pilot study^14^ including 50 consecutive adult outpatients (25 in each arm) undergoing a contrast-enhanced abdominal CT scan. In the pilot study we randomised positive (50 ml Télébrix Gastro in 950 ml water) and water only as oral contrast (1000 ml water only). Two radiologists independently rated scan quality and diagnostic confidence by a validated 5-point scale system. Almost all quality and diagnostic confidence scores were comparable between both observers and between both groups. One observer scored the diagnostic confidence of the gastrointestinal system as less than good in 10 of the patients receiving water only as oral contrast. Patients’ discomfort in this pilot study was assessed by a questionnaire. Although not statistically significant, Télébrix Gastro was more unpleasant for patients (n = 16, severe/mild/less than good) in comparison with water only (n = 12, severe/mild/less than good). Radiation exposure was extracted from dose reports. In the Télébrix Gastro group, the mean total DLP was 719.3 ± 245.7 mGy*cm^2^ and in the water only group 686.0 ± 206.9 mGy*cm^2^ (p = 0.62). The mean CTDI_vol_ was 11.1 ± 3.7 mGy and 9.8 ± 2.6 mGy respectively (p = 0.20). Although not significant, there was a trend towards higher values in the positive oral contrast group.

The pilot study showed that oral preparation with water only was just as sufficient and safe as positive oral contrast preparation with Télébrix Gastro diluted in water. To validate the hypothesis that water only as oral contrast is non-inferior to positive oral contrast preparation in abdominal CT, we performed a larger prospective study.

The aim of this prospective study was to compare the image quality rating, diagnostic confidence per structure in the abdomen, overall diagnostic confidence to address the clinical question and radiation exposure between water only and positive contrast (Télébrix Gastro diluted in water) as oral contrast agent in outpatients undergoing abdominal CT.

## Results

### Patient population and selection

**Figure 1:**
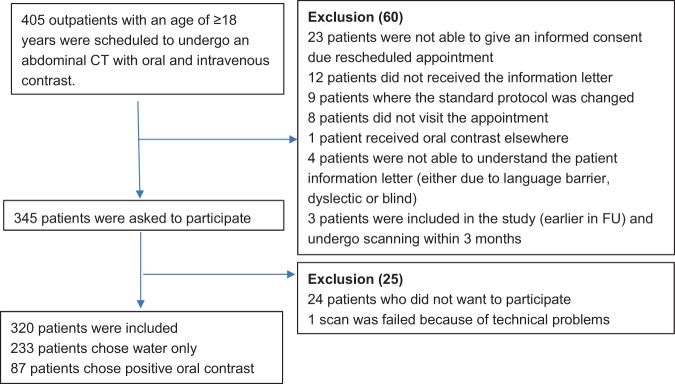
Selection and inclusion of patients.

Four hundred five (405) outpatients (age > 18 years) were scheduled to undergo an abdominal CT scan with oral and intravenous contrast. Sixty (60) patients were excluded due to various reasons (Fig. 1). 345 patients were asked to participate, of whom 24 did not want to participate. One patient was excluded due to technical problems. Finally, 320 patients were included and 233 (72.8%) chose water only as an oral contrast preparation. The remaining 87 patients (27.2%) chose positive oral contrast.

### Patients and CT characteristics

All patient baseline characteristics (Table 1) were comparable between both groups (all p-values > 0.05), except the height, which was p = 0.049. The same accounts for the CT characteristics (p > 0.05).

**Table 1:** Baseline patients and CT characteristics.

	Water only as oral contrast:1000 ml water (n = 233)	Positive oral contrast: 50 ml Télébrix Gastro diluted in 950 ml water (n = 87)	p-values
Sex distribution (male: female)	130:103	40:47	0.117
Age in years* (mean ± SD)	62.7 ± 12.17	62.9 ± 13.78	0.895
Height in cm* (mean ± SD)	174.3 ± 9.82	171.8 ± 9.69	0.049
Weight in kg* (mean ± SD)	78.0 ± 16.71	75.2 ± 14.98	0.178
BMI (kg/m^2^)* (mean ± SD)	25.7 ± 5.00	25.4 ± 4.15	0.609
Patient spectrum	Oncology: n = 200 (85.8%)	Oncology: n = 71 (81.6%)	0.124
	Haematology: n = 12 (5.2%)	Haematology: n = 10 (11.5%)	
	Others: n = 21 (9.0%)	Others: n = 6 (6.9%)	
Region scanned	Neck/Chest/Abdomen: n = 43 (18.5%)	Neck/Chest/Abdomen: n = 23 (26.4%)	0.200
	Chest/abdomen: n = 147 (63.0%)	Chest/abdomen: n = 46 (52.9%)	
	Abdomen: n = 43 (18.5%)	Abdomen: n = 18 (20.7%)	
CT scanner	64 slice scanners: n = 202 (86.7%)	64 slice scanners: n = 80 (92.0%)	0.196
	128 and 2*192 slice scanner: n = 31 (13.3%)**	128 and 2*192 slice scanner: n = 7 (8.0%)**	

*Age, height, weight, BMI and time interval were normally distributed. **Due to the low number of patients scanned on the 128 and 2*192 slice scanners, these data were combined.

### Radiation exposure

Although not significant, all medians (except CTDI_vol_ in the chest/abdomen region) were higher in the positive oral contrast group scanned by 64 slice scanners (Table 2). The medians of the total DLP and CTDI_vol_ of the different regions scanned by the 128 and 2*192 slice scanners seem comparable between the two oral contrast groups (Table 3), but this might be explained by the low number of patients.

**Table 2:** Radiation exposure in patients per region scanned by 64 slice scanners.

	Water only as oral contrast:1000 ml water(n = 199)*	Positive oral contrast: 50 ml Télébrix Gastro diluted in 950 ml water(n = 79)*	p-values
Total DLP in milligray*centimeters (median + range)	Neck/Chest/Abdomen (n = 37): 775.4 (420.0–1383.6)	Neck/Chest/Abdomen (n = 21) 815.4 (407.9–1167.8)	0.994
	Chest/abdomen (n = 125): 722.1 (327.3–1547.5)	Chest/abdomen (n = 41): 725.6 (328.0–1442.4)	0.877
	Abdomen (n = 37): 509.5 (329.4–1563.0)	Abdomen (n = 17): 650.2 (279.8–1035.2)	0.703
CTDI_vol_ in milligray (median + range)	Neck/Chest/Abdomen (n = 37): 9.11 (5.18–17.32)	Neck/Chest/Abdomen (n = 21): 9.51 (5.01–13.06)	0.815
	Chest/abdomen (n = 125): 10.53 (4.90–21.12)	Chest/abdomen (n = 41): 10.27 (5.81–19.85)	0.863
	Abdomen (n = 37): 10.31 (6.26–26.81)	Abdomen (n = 17): 11.40 (6.35–18.61)	0.485

All data were non-normally distributed and therefore Mann-Whitney test was performed to compare the distribution between the two arms. *Data on 4 patients were not taken into account, as they did undergo additional phase scanning.

**Table 3:** Radiation exposure in patients per region scanned by 128 and 2*192 slice scanners.

	Water only as oral contrast: 1000 ml water (n = 31)	Positive oral contrast: 50 ml Télébrix Gastro diluted in 950 ml water (n = 7)	p-values
Total DLP in milligray*centimeters (median + range)	Neck/Chest/Abdomen (n = 6) 575.6 (367.0–652.5)	Neck/Chest/Abdomen (n = 1) 533.1*	1.000
	Chest/abdomen (n = 20) 422.3 (214.7–755.1)	Chest/abdomen (n = 5) 415.0 (242.1–566.6)	0.530
	Abdomen (n = 5) 278.2 (213.2–462.7)	Abdomen (n = 1) 266.2*	0.667
CTDI_vol_ in milligray(median + range)	Neck/Chest/Abdomen (n = 6) 7.02 (4.58–8.43)	Neck/Chest/Abdomen (n = 1) 6.13*	0.857
	Chest/abdomen (n = 20) 6.12 (4.36–11.88)	Chest/abdomen (n = 5) 6.70 (3.66–8.10)	0.921
	Abdomen (n = 5) 6.33 (4.96–8.37)	Abdomen (n = 1) 5.30*	0.667

All data were non-normally distributed and therefore Mann-Whitney test was performed to compare the distribution between the two arms. *n = 1, therefore no range can be given.

### Image quality of the abdomen

**Figure 2:**
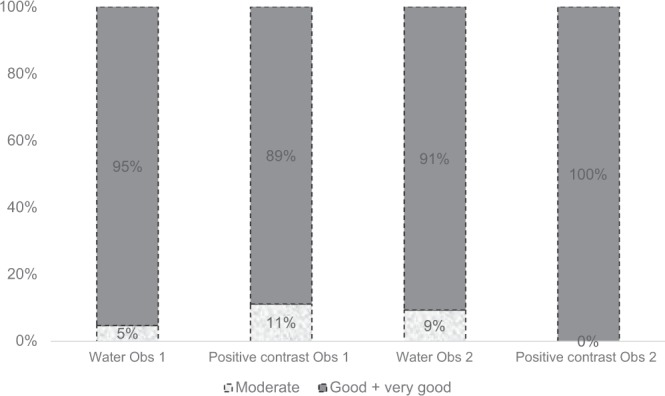
The quality of de scans assessed by observer 1 and observer 2.

**Figure 3:**
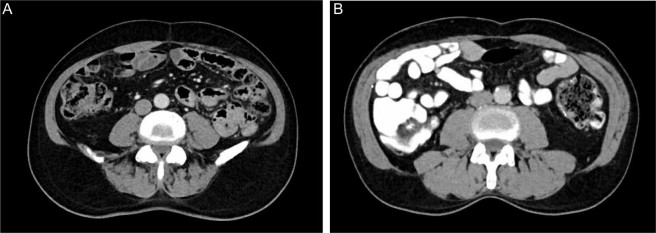
CT images of two patients with either water only or Télébrix Gastro as oral contrast. (**A**) shows an axial image of an abdominal CT of a 72-year-old man with metastatic renal cell carcinoma where the gastrointestinal tract was filled with water only (grey lumen). (**B**) shows an axial image of an abdominal CT of a 65-year-old man colorectal liver metastasis where the gastrointestinal tract was filled with Télébrix Gastro (white lumen).

The agreement between the observers was 86.9% (53/61). There was no difference between the water only and positive contrast for both observer 1 and observer 2 (p-values were respectively 0.574 and 0.310) (Figs. 2 and 3).

### Diagnostic confidence per structure of abdomen

#### Gastrointestinal system

**Figure 4:**
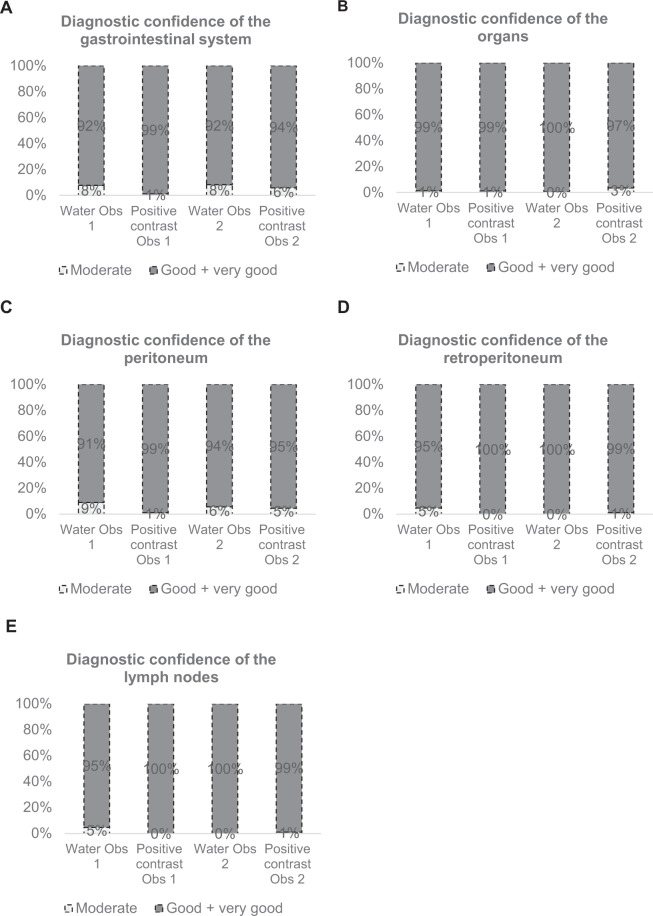
The diagnostic confidence of the different regions by observer 1 and observer 2.

The agreement between the observers for the evaluation of the diagnostic confidence was 90.3% (289/320). Observer 1 scored significantly more scans as less than good in the water only group compared to the positive contrast group (18/233 vs 1/87); p = 0.031. Observer 2 scored comparable diagnostic confidence of the gastrointestinal system between water only and the positive contrast groups (p = 0.634) (Fig. 4a).

#### Organs

The agreement between the observers for the evaluation of the diagnostic confidence was 97.5% (312/320). The diagnostic confidence was comparable between the water only and positive contrast groups, as assessed by observer 1 (p = 1.000) and observer 2 (p = 0.063) respectively (Fig. 4b).

#### Peritoneum

The agreement between the observers for the evaluation of the diagnostic confidence was 90.9% (291/320). Observer 1 scored significant more scans as less than good in the water only group compared to the positive contrast group (21/233 vs 1/87); p = 0.012. Observer 2 scored comparable diagnostic confidence of the gastrointestinal system between water only and the positive contrast groups (p = 1.000) (Fig. 4c).

#### Retroperitoneum

The agreement between the observers for the evaluation of the diagnostic confidence was 96.3% (308/321). Observer 1 scored significant more scans as less than good in the water only group compared to the positive contrast group (11/233 vs 1/87); p = 0.040. Observer 2 scored comparable diagnostic confidence of the gastrointestinal system between water only and the positive contrast groups (p = 0.272) (Fig. 4d).

#### Lymph nodes

The agreement between the observers for the evaluation of the diagnostic confidence was 96.3% (308/321). Observer 1 scored significant more scans as less than good in the water only group compared to the positive contrast group (11/233 vs 1/87); p = 0.040. Observer 2 scored comparable diagnostic confidence of the gastrointestinal system between water only and the positive contrast groups (p = 0.272) (Fig. 4e).

### Overall diagnostic confidence to address the clinical question

The agreement between the two observers for the evaluation of the overall diagnostic confidence was 96.3% (308/321; 307 fully addressing the clinical question and 1 partially addressing the clinical question). There were no scans where the clinical question could not be addressed. Observer 1 scored 8 scans as partially addressing the clinical question in patients with water only. Observer 2 scored 6 scans, 4 scans of patients with water only and 2 patients with positive oral contrast agent, as partially addressing the clinical question. In total, 13 patients, 11 with water only and 2 with positive oral contrast were rechecked by observer 3 (Table 4).

**Table 4:** Patients with partially assessed overall diagnostic confidence by either observer.

Patient number	Water only or positive contrast	Patient Spectrum	Age in years	Sex (male or female)	Observer 1	Observer 2	Observer 3
**1**	Water only	Oncology	79.3	Female	Partially addressed	Fully addressed	Fully addressed
**2**	Water only	Oncology	77.6	Male	Partially addressed	Fully addressed	**Partially addressed**
**3**	Water only	Oncology	63.7	Male	Partially addressed	Fully addressed	**Partially addressed**
**4**	Water only	Other	66.5	Male	Fully addressed	Partially addressed	**Partially addressed**
**5**	Water only	Oncology	64.8	Female	Fully addressed	Partially addressed	**Partially addressed**
**6**	Water only	Oncology	65.9	Male	Partially addressed	Partially addressed	**Partially addressed**
**7**	Water only	Other	71.7	Female	Partially addressed	Fully addressed	Fully addressed
**8**	Water only	Oncology	65.9	Female	Partially addressed	Fully addressed	Fully addressed
**9**	Water only	Oncology	81.5	Female	Fully addressed	Partially addressed	**Partially addressed**
**10**	Water only	Oncology	72.3	Female	Partially addressed	Fully addressed	Fully addressed
**11**	Water only	Other	68.9	Male	Partially addressed	Fully addressed	Fully addressed
**12**	Positive oral contrast	Other	49.7	Female	Fully addressed	Partially addressed	Fully addressed
**13**	Positive oral contrast	Oncology	78.4	Female	Fully addressed	Partially addressed	Fully addressed
					**8 of 13 partially addressed**	**6 of 13 partially addressed**	**6 of 13 partially addressed**

In 7 patients (5 with water only and 2 patients with positive oral contrast), the clinical question could be answered by observer 3. The remaining 6 scans were also scored as partially addressed by observer 3. A total of 6/233 vs 0/87 were scored as partially addressed (p = 0.259). Scans that partially addressed the clinical questions, were due to; 1) difficulties in evaluating peritoneal and/or omental depositions; 2) fistulas and liquid collections; and 3) difficulties in performing mass measurement if located close to or merged with the intestine.

## Discussions

### Summary

We found that outpatients undergoing abdominal CT scan preferred water only as oral contrast above the positive oral contrast. This does not influence image quality. One observer experienced slightly less confidence in the evaluation of the GI tract, peritoneum, retroperitoneum and lymph nodes; this might be explained by the lower experience of the observer.

In addition, the clinical question could be fully addressed in the majority of patients. Only 6/233 in the water only group vs 0/87 in the positive oral contrast group were scored as partially addressed. In none of the patients, could the clinical questions not be addressed. Scans that only partially addressed the clinical questions were due to the difficulties in evaluating peritoneal and/or omental depositions; fistulas and liquid collections; and mass measurement if located close to or merged with the intestine, due to missing positive oral contrast. There was no significant difference in radiation exposure between both groups. However, the positive oral group showed a tendency towards a higher radiation exposure.

### Compared to other studies

In several studies, the role of water only as oral contrast has been evaluated^7–10^.

In the study of Kammerer *et al*.^7^, mainly oncology patients (68%), with different types of oral preparation (positive contrast agent, n = 576; water only, n = 716; and no oral contrast, n = 716) were retrospectively evaluated. Delineation of the bowel was evident across all segments regardless of the type of oral contrast and a slight impairment (concerning diagnostic reliability) was observed in patients without the use of any oral contrast.

In the study of Buttigieg *et al*.^8^, 46 oncological patients who previously underwent abdominopelvic CT with positive oral contrast, were scheduled for follow-up and received water only as oral contrast (n = 25) or no oral contrast agent (n = 21). The data showed comparable image quality concerning the reproduction of abdominal structures, bowel discrimination, presence of artefacts, and visualization of the amount of intra-abdominal fat for the three protocols.

The study by Lee *et al*.^9^, with 103 patients who received both (water only and positive oral contrast) strategies also showed significantly better delineation of duodenal wall (p < 0.001), and overall visualisation of the duodenum (p = 0,001), using water only compared to positive oral contrast including Télébrix Gastro. Comparable results were observed for visualisation of the other abdominopelvic organs, wall delineation of the small bowel and contrast-associated artefacts.

Makarawo *et al*.^10^ studied image clarity and luminal distention in 66 patients who received both a pancreas protocol CT (PPCT) that uses oral water and abdominal conventional positive oral contrast scan. CT images were independently reviewed by two radiologists who scored the degree of hollow viscus distention and visualization of mural detail using a Likert 5-point scale. The PPCT had a better median score for organ clarity in the stomach and duodenum (P < 0.001) and better luminal distention in the stomach (P < 0.001), equal distention in the duodenum (P = 0.02), and slightly worse distention in the ileum (P = 0.02). The remaining bowel and organs were evaluated with no statistically significant difference in the ratings between the two protocols. They concluded that using present CT scan technology, water can be an effective contrast medium causing better or equal distention in the bowel and better or equal clarity than routine barium contrast.

However, all data of the above-mentioned studies^7–10^ was retrospectively obtained. In our previous prospective pilot study^14^ including 50 consecutive adult outpatients (25 in each arm), quality and diagnostic confidence scores were comparable between water and positive oral contrast. Positive oral contrast caused more discomfort (n = 16, severe/mild/less than good) in comparison with water only (n = 12, severe/mild/less than good).

Concerning the radiation exposure, one study by Wang *et al*.^11^ showed higher radiation exposure for scans performed with positive oral contrast than those with water as oral contrast in respectively 13.1 mGy and 11.8mGy (p = 0.003). In our previous study^14^, the mean total DLP was 719.3 ± 245.7 mGy*cm^2^ and 686.0 ± 206.9 mGy*cm^2^ (p = 0.62) in the Télébrix Gastro and water only respectively. The mean CTDI_vol_ was 11.1 ± 3.7 mGy and 9.8 ± 2.6 mGy respectively (p = 0.20). In this study, although not significant, there was also a trend towards higher values in the positive oral contrast group. The findings of this study concerning the image quality, diagnostic confidence and the diagnostic reliability (clinical question) and radiation exposure were in line with the results of all the above mentioned studies^7–11^.

### Strengths

To our knowledge this is the first large prospective study performed on this topic. The advice of oral contrast preparation dates back to the year 2000^15^. We did not only evaluate the image quality and diagnostic confidence, but also the overall diagnostic confidence and the radiation exposure. Two abdominal radiologists with different levels of experience reviewed all of the scans independently.

### Limitations

There were some limitations in the present study. This study is not a randomised controlled trial but a prospective case-control study. This design was selected, as both types of contrast agents were used to a different extent in the general practice and we also aimed to study the preference of patients. Although patients were free to choose between water only and Télébrix Gastro as oral contrast, we do not think this generated a high risk of bias, as baseline characteristics were comparable. We excluded patients with a primary gastro-intestinal indication. It is known that opacification of the gastrointestinal (GI) system by water only effects the diagnosis of the GI system^12,13^. However, the patient population in our study consist of mainly oncological patients where the focus lies on metastases and not on the GI tract. For the specific interpretation of GI systems (for e.g. polyps or Crohn’s disease), regular CT scans are not sufficient and more specific imaging is needed, such as CT enterography or colography. Finally, later emerging contra-indications like diarrhea, nausea or vomiting were not taken into consideration in this study^16^. The current study focuses only on subjective grading of image quality and diagnostic confidence, and does not test miss-rates for disease. Ultimately, the miss-rate of bowel and peritoneal disease is the most important end point, and will require larger numbers of scans and appropriate follow-up studies in the future.

## Conclusions

We found that abdominal CT with water only has comparable diagnostic confidence as abdominal CT with positive oral contrast in the majority of outpatients. Therefore, water only can replace positive oral contrast in the standard CT protocol for the majority of outpatients scheduled to undergo an abdominal CT.

## Materials and Methods

This study was conducted according to the principles of the Declaration of Helsinki (64^th^ WMA General Assembly, Fortaleza, Brazil, October 2013) and a waiver concerning the Medical Research Involving Human Subjects Act (WMO) regulation was obtained. This study is reported according to Strobe (Strengthening the Reporting of Observational Studies in Epidemiology) guidelines^17^.

### Population

Consecutive adult outpatients that were scheduled to undergo an abdominal CT scan with oral and intravenous contrast at the department of Radiology and Nuclear Medicine between June 2018 and September 2018 were included. The indications for abdominal CT scan with oral contrast were: follow-up oncology, chronic pancreatitis, pseudocyst, follow-up hepatopancreaticobiliary (HPB) surgery and hematology. Inclusion criteria were: (1) patients scheduled to undergo an abdominal CT scan (with or without neck and chest scan) with oral and intravenous contrast; (2) outpatients and (3) age ≥18 years. Exclusion criteria were: (1) patients undergoing CT for research purposes; (2) patients who were not able to drink; and (3) patients requiring positive oral contrast for evaluation of the gastrointestinal/intraluminal tract (primary staging and response monitoring of colon tumour, staging of inflammatory bowel disease, evaluation of anastomotic leaks, evaluation of gastrointestinal stromal tumour) and patients with complex problems (cause unknown).

### Procedure

Patients scheduled to undergo contrast-enhanced abdominal CT scan and fulfilling inclusion criteria received a letter 5–8 days before the scheduled CT scan. Patients were asked to choose between water only as oral contrast (1000 ml water) or positive oral contrast (50 ml Télébrix Gastro (Guerbet, Villepinte, France) diluted in 950 ml water), as both methods are general accepted methods in The Netherlands. Written informed consent for the use of data was obtained before the CT scan started. Patients who chose water only were instructed to drink this volume in 45 minutes. The patients who chose positive oral contrast (50 ml Télébrix Gastro + 950 ml water) were instructed to drink within 60 minutes as standard protocol. No adjustments were made concerning intravenous administration of the contrast agent.

### CT acquisition

CT scans were performed according to our routine protocol, using 4 different CT systems. Two 64 slice systems (SOMATOM Sensation, Siemens Healthcare, Erlangen, Germany and Philips Brilliance, Philips Medical Systems, Best, The Netherlands), one 128 slice system (SOMATOM Definition AS + , Siemens Healthcare, Erlangen, Germany) and one dual source 2*192 slice system (SOMATOM Force, Siemens Healthcare, Erlangen, Germany) were used. Iomeron (300 mg I/ml, Bracco UK limited, High Wycombe, UK) was used as intravenous contrast agent. The intravenous scan protocol for the SOMATOM Force contains 80 ml Iomeron. The other three scanners (Sensation, Definition AS + , and Brilliance) used 100 ml Iomeron.

### Sample size calculation

In the pilot study^14^, the percentage of overall diagnostic confidence was rated as good in 98.4% and 94.4% patients with respectively positive oral contrast and water only. Based on these findings and a non-inferiority design, we had to include at least 210 patients (water only group) with a power of 90% (90% sure) and an upper limit of one-sided 95% confidence interval to exclude a difference in favour of the standard group of more than 4%.

### Data-extraction

Age, sex, height, weight, patient spectrum (oncological, haematological or other), scanned regions and CT scanner system were extracted/reported.

Radiation exposure measures such as CTDI_vol_ (volume CT dose index) and DLP (Dosis Length Product) were extracted from radiation exposure reports of each scanner. In our institution, a dose report is electronically captured for all CT exams with CTDI_vol_ and DLP^18^.

### Data evaluation

Two abdominal radiologists, with respectively 6 years (observer 1) and 19 years (observer 2) experience in evaluating abdominal scans, reviewed each image set independently. Images were presented in a random order and blinding of the images was not necessary as high attenuation images with positive oral contrast were being compared to images with water only as oral contrast and this could not be concealed. They rated the image quality of the abdomen, the diagnostic confidence per structure in the abdomen and finally an overall diagnostic confidence to address the clinical question.

#### Image quality of the abdomen

Image quality of the abdomen was assessed according to an ordinal rating scale of five response categories; (1) very poor; (2) poor; (3) less than good; (4) good and; (5) very good, adapted from Båth and Månsson^19^.

#### Diagnostic confidence per structure in the abdomen

The diagnostic confidence per system was also rated according to the same 5-point scale. The following five systems were rated;(1) gastrointestinal system (stomach, duodenum, jejunum, ileum, colon and appendix); (2) organs (liver, spleen, pancreas, adrenals, gallbladder, kidneys including ureters, bladder, ovaries, uterus or prostate); (3) peritoneum; (4) retroperitoneum; and (5) lymph nodes^9^.

#### Overall diagnostic confidence to address the clinical question

An overall diagnostic confidence was assessed using a 3-point scale; 1) not able; 2) partially able; and 3) fully able to address the clinical question. Any scan scored as partially able to address the clinical question by one of the two radiologists was checked independently by a third abdominal radiologist (observer 3) with experience in abdominal CT reporting of 7 years. The abdominal radiologist had to score the scans by choosing; 1) partially able; and 2) fully able to address the clinical question.

### Statistical analysis

#### Patients and CT characteristics

Baseline data was summarized using descriptive statistics. Both groups were compared using the Student’s t-test statistic (normal distributed continuous data), Mann-Whitney U-test (non-normal distributed continuous data) or chi-squared test for categorical data. And the medians and ranges of the total DLP and CTDI_vol_ of the different regions between the two groups were compared by using the Mann-Whitney test.

#### Study parameters

The results of image quality and diagnostic confidence per structure between the two groups were expressed as proportion and corresponding 95% confidence intervals. These data were categorised in three categories (1 and 2, 3, 4 and 5) and null hypothesis of no difference was evaluated by chi-squared test for trend, due to the ordinal character of the data. Agreement between observers was expressed as percentages.

The results on overall diagnostic confidence (not able, partially able or fully able to address the clinical question) were expressed as proportion of corresponding 95% confidence interval and evaluated by chi-squared test. Agreement between the observers was expressed as percentages.

## Data Availability

The datasets used and/or analysed in this study are available from the corresponding author on reasonable request.
